# Influence of Paclitaxel and Doxorubicin Therapy of ßIII-Tubulin, Carbonic Anhydrase IX, and Survivin in Chemically Induced Breast Cancer in Female Rat

**DOI:** 10.3390/ijms22126363

**Published:** 2021-06-14

**Authors:** Alena Pastornická, Silvia Rybárová, Slávka Drahošová, Jozef Mihalik, Andrea Kreheľová, Andriana Pavliuk-Karachevtseva, Ingrid Hodorová

**Affiliations:** 1 Department of Anatomy, Medical Faculty, Šafárik University, Šrobárova 2, 041 83 Košice, Slovakia; alena.pastornicka@student.upjs.sk (A.P.); silvia.rybarova@upjs.sk (S.R.); jozef.mihalik@upjs.sk (J.M.); andrea.krehelova@student.upjs.sk (A.K.); andriana.pavliuk-karachevtseva@upjs.sk (A.P.-K.); 2 Department of Pathological Anatomy, Jessenius Faculty of Medicine, Comenius University, Kollárova 2, 036 59 Martin, Slovakia; slavka.drahosova@hermeslab.sk

**Keywords:** βIII-tubulin, breast cancer, carbonic anhydrase IX, doxorubicin, paclitaxel, survivin, rats

## Abstract

Breast cancer is the most common cancer in females. The aim of this study was to determine the effect of paclitaxel (PTX) and doxorubicin (DOX) therapy on the βIII-tubulin, carbonic anhydrase IX (CA IX), and survivin expression in chemically-induced rat mammary tumors. Animals with induced mammary carcinogenesis were randomly divided into treatment groups and an untreated group. The total proportion of tumors, the proportion of carcinoma in situ (CIS), and invasive carcinoma (IC) were evaluated. Protein expression in tumor tissue was determined using IHC. Statistical analysis of the data, evaluated by Fisher-exact test and unpaired *t*-test. Significantly increased levels of proteins in the tumor cells were confirmed using the IHC method for all studied proteins. The expression of βIII-tubulin, CA IX, and survivin increased significantly after treatment with both cytostatics (PTX and DOX). Depending on the type of tumor, a significant increase in all proteins was observed in IC samples after PTX treatment, and CA IX expression after DOX treatment. In CIS samples, a significant increase of βIII-tubulin and survivin expression was observed after a DOX treatment. The results suggest that βIII-tubulin, survivin, and CA IX may be significant drug resistance markers and the clinical regulation of their activity may be an effective means of reversing this resistance.

## 1. Introduction

Breast cancer is the most common cancer in women today. It is the fifth leading cause of death (6.6%) and also causes the highest number of deaths (approximately 15%) related to women’s cancer [1]. Anthracyclines, such as doxorubicin and epirubicin, taxanes, including paclitaxel and docetaxel, and fluorouracil and cyclophosphamide are current first-line therapeutics for the combined adjuvant treatment of breast cancer [2]. Paclitaxel (PTX), a diterpene alkaloid, is commonly used to treat breast cancer. Microtubules are the cellular target of PTX. The principal actions of PTX are induction mitotic arrest through polymerization and stabilization of microtubules and the induction of cell death by apoptosis [3]. Doxorubicin (DOX) is an anthracycline drug commonly used to treat breast, lung, stomach, ovarian, and many other types of cancer. The main effects of anthracyclines are DNA intercalation, inhibition of topoisomerase II, and the formation of free radicals. As a result, lipid peroxidation, cell membrane damage, DNA damage, and oxidative stress occur, leading to apoptosis [2]. Resistance to these chemotherapeutics is a primary barrier to effective treatment because resistance to apoptosis is an important mechanism for tumor cells to contribute to cancer progression.

Many studies have examined the prognostic association of the expression of various proteins in tumor tissues with the response to treatment. The development of tolerance to taxanes is largely attributed to microtubule changes and, in particular, the overexpression of specific ß-tubulin isotypes [4].

ß-tubulin exists in the form of several isotypes that are differentially expressed in normal and neoplastic cells and differ in their ability to bind to drugs. In particular, the βIII isotype is overexpressed in many aggressive and metastatic tumors and may serve as a prognostic marker in certain types of cancer [5]. However, evidence relating to its role in taxane resistance is very controversial. According to several authors, high βIII-tubulin expression in breast cancer was associated with susceptibility to taxane-based chemotherapy, not resistance [6,7]. Based on this, it is possible to assume that βIII-tubulin is not a predictive biomarker of taxane resistance, and, instead, βIII-tubulin is a pure prognostic biomarker [7]. In contrast, Yuan et al. [8] investigated the relationship between βIII-tubulin and survivin expression in invasive breast cancers and the chemotherapeutic effects of docetaxel. The response to treatment was significantly lower in patients with high βIII-tubulin and survivin expression than in patients with negative expression of these proteins (25.0% vs. 73.91%, *p* < 0.05). Their results suggest that high expression of both proteins may be responsible for docetaxel drug resistance in patients with advanced breast cancer.

Survivin, like βIII-tubulin, is considered a potential target for the treatment of cancer, mainly due to its dual role. It acts as a suppressor of apoptosis and also plays an important role in cell proliferation. Survivin dysfunction leads to cell cycle defects, including disruption of cytokinesis, centrosome dysregulation, and multinucleated cell formation [9]. In addition, survivin also plays an essential role in the angiogenesis process, as has been demonstrated in vitro. The significance lies in its ability to protect endothelial cells from apoptosis. Through this protection of newly formed blood vessels, it can indirectly contribute to tumor growth [10]. Survivin is undetectable in most proliferating, fully differentiated cells. In contrast, its high expression is confirmed in many types of malignant cells. In tumor cells, survivin accumulates and localizes in the cytoplasm, cell nucleus, and mitochondria. Khan et al. [11] found that the various functions that survivin performs appear to be related to where it is located. In the nucleus, it plays a role in the regulation of mitosis, while in the cytoplasm it modulates the inhibition of apoptosis. It is believed that its presence in the extracellular environment may increase tumor aggressiveness and also reduce or inhibit treatment efficacy. Hypoxia can also cause therapeutic resistance.

The expression of CA IX proteins is induced by hypoxia in various tumors. Through their ability to regulate pH and create an acidic environment they increase the chance of tumor cell survival in hypoxia conditions and provide an increased ability to migrate [12]. Under physiological conditions, CA IX expression is usually restricted to the gastrointestinal tract to maintain an acidic pH [13]. However, it is very often and strongly expressed in tumors. Several reviews have summarized the importance of CA IX as a promising biomarker for tumorigenesis. CA IX also provides resistance to treatment including chemotherapy, radiotherapy, and antiangiogenic therapy. Thus CA IX is a clinically relevant biomarker and potential anti-cancer target [14].

In the present study, we investigated whether levels of βIII-tubulin, CA IX, and survivin could change in induced breast cancer in rats as a result of treatment with PTX and DOX.

## 2. Results

### 2.1. Histopathology

To detect the tumor stage, the slides were stained by hematoxylin-eosin [15]. We divided the tumors as follows:

A1. Treatment with paclitaxel:
  1. Non-treated group: n = 13 tumors.
  2. Treated group: n = 49 tumors
B1. Stage of tumor:
  1. Carcinoma in situ (CIS): n = 32 tumors.
  2. Invasive carcinoma (IC): n = 30 tumors.
A2. Treatment with doxorubicin:
  1. Non-treated group: n = 29 tumors.
  2. Treated group: n = 34 tumors
B2. Stage of tumor:
  1. Carcinoma in situ (CIS): n = 43 tumors.
  2. Invasive carcinoma (IC): n = 20 tumors.

### 2.2. Immunohistochemistry—ßIII-Tubulin

**Figure 1:**
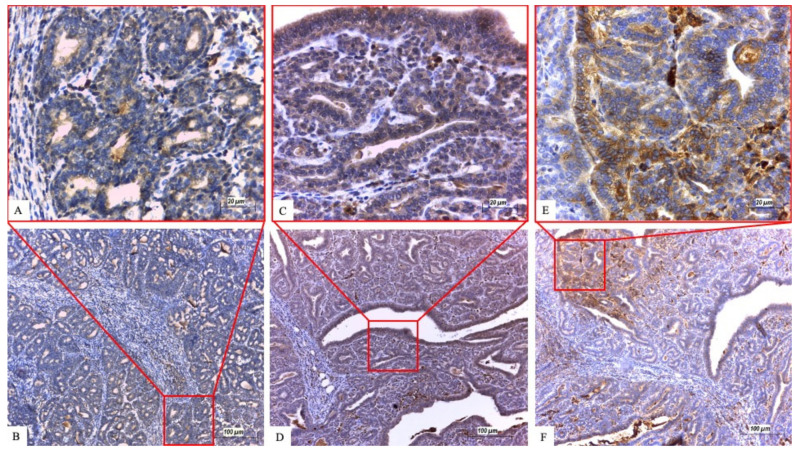
Immunohistochemical expression of proteins in invasive carcinoma. (**A**,**B**) ßIII-tubulin. (**C**,**D**) CA IX. (**E**,**F**) Survivin. Scale bars (**A**,**C**,**E**) 20 µm; (**B**,**D**,**F**) 100 µm. The upper images show the area with the red rectangle on lower images at higher magnification.

**Figure 2:**
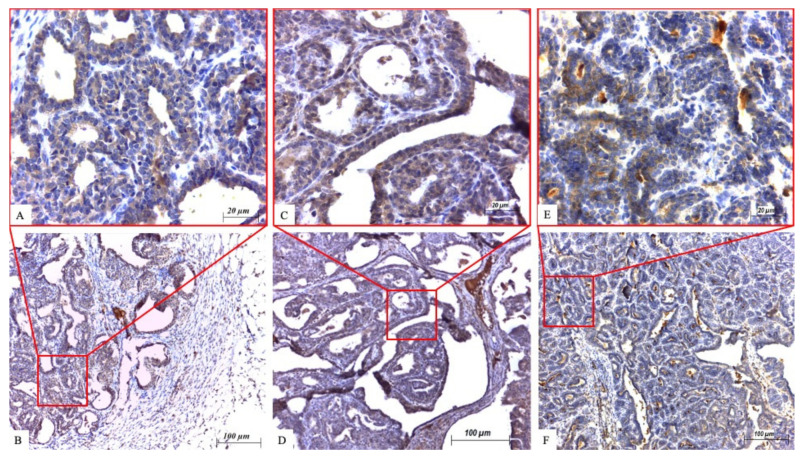
Immunohistochemical expression of proteins in carcinoma in situ. (**A**,**B**) ßIII-tubulin. (**C**,**D**) CA IX. (**E**,**F**) Survivin. Scale bars (**A**,**C**,**E**) 20 µm; (**B**,**D**,**F**) 100 µm. The upper images show the area with the red rectangle on lower images at higher magnification.

ßIII-tubulin showed cytoplasmic positivity (Figure 1A,B; Figure 2A,B). In the group of treated animals, 34 IC tumors developed during the experiment. In the doxorubicin group eight tumors were ßIII-tubulin positive (88.9%) and one sample showed negative ßIII-tubulin reactivity (11.1%). In the paclitaxel group, 22 out of 25 tumors were ßIII-tubulin positive (88%). The treated group with 48 CIS tumors contained 19 (79.2%) ßIII-tubulin positive samples in the paclitaxel group and 24 (96%) positive samples in the doxorubicin group.

In the group of non-treated animals, 16 IC tumors were found. From the paclitaxel group, four of them were ßIII-tubulin negative (80%) and one sample was ßIII-tubulin positive (20%). Five tumors from doxorubicin-treated rats were ßIII-tubulin negative (45.5%), while six tumors were ßIII-tubulin positive (54.5%). Samples of CIS showed two (25%) negative tumors in the paclitaxel group and 10 (55.5%) negative tumors in the doxorubicin group (Table 1). A comprehensive evaluation of staining revealed that up to 83.7% of tumors showed ßIII-tubulin positivity after paclitaxel treatment and 94.1% of tumors were positive after doxorubicin treatment (Table 1).

Statistical analysis in the paclitaxel group revealed that IC treated tumors differed significantly from IC non-treated tumors (*p* < 0.05). However, when CIS treated tumors were compared with CIS non-treated tumors the results did not show statistical significance (*p* > 0.05). In the doxorubicin group, the IC treated tumors did not show statistical significance compared to untreated IC tumors but statistical significance was confirmed (*p* > 0.05) in the comparison of CIS tumors. The evaluation of staining in untreated compared to treated tumors revealed that IHC expression of βIII-tubulin increased significantly (*p* > 0.05) in breast tumors after treatment with both paclitaxel and doxorubicin (Table 1).

### 2.3. Immunohistochemistry—CA IX

CA IX was immunohistochemically expressed in the cytoplasm when positive (Figure 1C,D; Figure 2C,D). In the IC subgroup of paclitaxel-treated rats, 23 (92%) CA IX positive and only 2 (8%) CA IX negative tumors were found. In the doxorubicin group, eight positive tumors (88.9%) of the nine IC tumors were found. From the 24 CIS tumors in the paclitaxel group, 15 (62.5%) were CA IX positive and 19 (76%) samples showed positive CA IX reactivity in the doxorubicin group.

IHC detection of CA IX protein in the non-treated CIS subgroup had four (50%) and seven (38.9%) negative samples in the paclitaxel and doxorubicin group, respectively, while the IC subgroup had three (60%) negative samples in the paclitaxel group and seven (63.6%) negative samples in the doxorubicin group (Table 2). All in all, in the paclitaxel group, 77.6% of the tumors were positive and 79.4% of the tumors were positive in the doxorubicin group (Table 2).

The statistical evaluation showed that IC treated tumors in the paclitaxel and doxorubicin groups only differed significantly from IC non-treated tumors (*p* < 0.05). The difference in CA IX expression between CIS treated vs. CIS non-treated tumors was statistically non-significant (*p* > 0.05) in both groups. Similar to the ßIII-tubulin, expression of CA IX in the paclitaxel and doxorubicin treated groups, staining significantly increased in tumor samples compared with non-treated animals (Table 2).

### 2.4. Immunohistochemistry—Survivin

Survivin was expressed in the cytoplasm of tumor cells (Figure 1E,F; Figure 2E,F). IHC detection of survivin in the paclitaxel-treated IC subgroup had 19 (76%) positive samples. Out of all doxorubicin-treated IC samples (nine), eight (88.9%) were positive, while only one sample showed negative survivin reactivity. Substantial differences between survivin negative and positive samples were demonstrated in the CIS of doxorubicin-treated rats. Survivin negative immunoreactivity was found in only two samples while 23 (92%) tumors exhibited survivin positivity. In the paclitaxel group, 24 CIS tumors were found in total and 14 (58.3%) were survivin reactive (Table 3). In the overall evaluation, 67.3% positive tumors were found in the paclitaxel group and the confirmed protein expression in the doxorubicin group was 91.2% (Table 3).

The statistical analysis showed that IC tumors treated with paclitaxel were significantly different from non-treated tumors (*p* < 0.05) while IC tumors treated with doxorubicin were not statistically significantly different from non-treated tumors. The difference in survivin expression between CIS paclitaxel treated vs. CIS non-treated tumors was statistically non-significant (*p* > 0.05). On the other hand, statistical comparison of CIS doxorubicin-treated and untreated tumors showed statistical significance (*p* < 0.05). The overall expression of survivin in the group of non-treated compared with treated animals significantly increased staining in tumor samples (*p* < 0.05) after both paclitaxel and doxorubicin treatment (Table 4).

## 3. Discussion

This study demonstrates that diffuse cytoplasmic overexpression of βIII-tubulin, survivin, and CA IX in tumor tissues of mammary gland carcinoma is related to chemosensitivity.

In general, βIII-tubulin plays a crucial role in the cell cycle, cell proliferation, differentiation, and tumorigenesis due to its unique microtubule depolymerization activity. In addition, it can control the cell cycle and thereby alter cell proliferation, differentiation, and other biological behaviors [6]. According to Xiang et al. [16], βIII-tubulin expression changes the features of different endogenous microtubules in tumor cells. These changes cause reduced sensitivity of the binding between the drug and βIII-tubulin dimers. Additionally, high expression of βIII-tubulin reduces the rate of microtubule polymerization; this property could be responsible for resistance to taxanes, which are drugs that promote tubulin assembly.

Several studies have debated the possible correlation between taxane resistance and high ßIII-tubulin expression. Expression of the neuron-specific βIII-tubulin isotype correlated with taxane resistance in vitro and in vivo and has been extensively studied as a clinical biomarker of taxane efficacy [17]. Our study revealed that βIII-tubulin is frequently overexpressed in mammary gland carcinoma after PTX and DOX treatment. We were able to demonstrate the presence of ßIII-tubulin in 43.8% (7/16) of IC and 53.8% (14/26) of IS in the non-treated group, while 88% (22/25) of IC and 79.2% (19/24) of CIS were positive in the group treated with PTX and 88.9% (8/9) of IC and 96% (24/25) of CIS were positive in the group treated with DOX. Statistical analysis of the paclitaxel group revealed that IC treated tumors differed significantly from IC non-treated tumors (*p* < 0.05) and, in comparison to CIS tumors from the doxorubicin group, we also found that statistical significance was confirmed (*p* > 0.05). These results indicate that βIII-tubulin expression may be an early event in the malignant process after doxorubicin therapy. In addition to cytoplasmic activity, the stromal activity of the protein was detected. According to Person et al. [18], βIII-tubulin is poorly expressed in healthy mammary glands in fibroblasts. According to Dozier et al. [19], the expression of βIII-tubulin in the tumor tissue was observed mainly in the form of diffuse staining of stromal tissue. In some places, irregular staining was observed, which the authors attribute to the cross-section of nerve fibers. The presence of ßIII-tubulin in nerve fibers is explained by its high specificity for neurons where it serves as a marker. Evaluation of the total stained tumors in our study revealed that up to 83.7% of tumors were positive for βIII-tubulin after paclitaxel treatment (*p* > 0.05) and 94.1% of tumors were positive after doxorubicin treatment (*p* > 0.05). These results are not consistent with the study of Yuan et al. [8] who observed βIII-tubulin positive cells in only 38.1% of cases (32/84) excised after taxane treatment. In contrast, Im et al. [20] found 60.7% (111/183) positive cases of breast cancer but the loss of βIII-tubulin expression was associated with aggressive behavior of tumor tissue treated with taxane-containing chemotherapy. Wang et al. [21] determined the expression of βIII-tubulin in breast cancer samples and the result of their analysis was an increase in expression after doxorubicin administration, which is consistent with our results. However, high expression significantly correlated with a higher probability of achieving a good response to chemotherapy. Xiang et al. [16] analyzed samples from 48 breast cancer patients who received neoadjuvant therapy containing taxanes. The expression of the βIII-tubulin protein before chemotherapy significantly affects the correlated treatment with the response rate. The overall response rate was 31.8% in the high expression group, while the rate was 84.6%. in the low βIII-tubulin expression group. All eight patients with complete pathological remission were in the low βIII-tubulin expression group. Based on these results the authors consider βIII-tubulin to be a suitable predictor of taxane chemosensitivity and also hypothesize that changes in expression levels during chemotherapy may be effective on resistance.

As previously mentioned, the results of this work show that βIII-tubulin is overexpressed in tumor tissue after cytostatic treatment. The results justify further studies considering the validation of this marker for its potential role as a predictive biomarker in certain types of breast cancer. Several studies have clearly indicated that the expression of βIII-tubulin can be induced in normal as well as neoplastic cells exposed to a toxic microenvironment characterized by hypoxia and nutrient deficiency [6].

Hypoxic cancer cells overexpress CA IX, which provides a tumor-favorable intracellular pH, contributing to stromal acidosis and facilitating tumor invasion and metastasis [22]. In recent years, these changes in the microenvironment have been considered potential therapeutic targets and key factors in the metastasis and recurrence of breast cancer [23]. Our study demonstrated the presence of CA IX in 37.5% (6/16) of IC and 57.7% (15/26) of IS in the non-treated group. Wykoff et al. [24] observed CA IX activity in 38% (11/29) of cases of IC and in 59% (23/39) of cases of CIS. These results are consistent with our results in the untreated group of animals. All samples in their study showed membrane positivity. As CA IX is a type I transmembrane protein it is mostly detected on the plasma membrane of tumor cells in immunohistochemistry. This is not consistent with our results where very few tumor cells showed membrane activity. Almost all positive tumor cells expressed CA IX in the cytoplasm. Positivity was also observed in the tumor stroma. Since CA IX may be endocytosed, cytoplasmic positivity may also be observed. Cellular receptors can signal not only from the cell surface but also from the endosomes. It can also occur in the cellular components of the tumor stroma. This stromal signal represents either the induction of endogenous CA IX in cancer-associated fibroblasts or the extracellular domains of CA IX released from tumor cells and bound to the surface of immune cells or potentially endocytosed in these cells [25]. CA IX is thought to promote the resistance of tumor cells to various treatments, including chemotherapy, radiotherapy, and antiangiogenic therapy. In patients with invasive breast cancer immunohistochemical expression of CA IX correlated with poorer relapse-free survival, as well as overall survival. This poor prognosis may be due to treatment-mediated hypoxia resistance or radiotherapy [26]. After treatment with cytostatics in our study, the presence of CA IX was demonstrated in 92% (23/25) of IC and 62.5% (15/24) of CIS treated with PTX and 88.9% (8/9) of IC and 76% (19/25) of CIS treated with DOX. The statistical evaluation showed that IC treated tumors in the paclitaxel and doxorubicin groups only differed significantly from IC non-treated tumors (*p* < 0.05). The difference in CA IX expression between CIS treated vs. CIS non-treated tumors was statistically non-significant (*p* > 0.05) in both groups. These results suggest that CA IX expression may be a significant predictor of tumor invasiveness. In the paclitaxel group, 77.6% of tumors were positive and 79.4% of tumors were positive in the doxorubicin group. Similar to the ßIII-tubulin expression of CA IX in the paclitaxel and doxorubicin non-treated groups, staining significantly increased in tumor samples after treatment compared with treated animals. The overexpression of CA IX in mammary tumors that was demonstrated in this study after treatment with cytostatics is likely to indicate an increase in drug-induced resistance.

Survivin, also known as BIRC5, is essential for cell division and can inhibit apoptosis and increase angiogenesis. It is normally only expressed in actively propagating cells, but its increased expression has been shown in almost all types of malignancies. In addition to tumor cells, survivin has also been shown in autoimmune diseases such as rheumatoid arthritis and multiple sclerosis, where its cytokine-dependent expression correlates with decreased apoptosis and inflammation. For oncologists looking for a new targeted treatment, the proteins needed for cell proliferation and the proteins related to apoptosis are at the top of the most-wanted list. Therefore, as a protein essential for mitosis and capable of inhibiting apoptosis, survivin is a promising protein for targeted therapy [27]. We detected survivin expression in 50% (8/16) of IC and 38.5% (10/26) of IS in the non-treated group. When comparing all 42 tumors, regardless of tumor type, the expression of survivin was observed in 42.9% (18/42) of tumors. These results are not consistent with other studies. Kennedy et al. [28] found a 60% positivity for survivin in mammary tumors (176/293 cases). As many as 117 tumors did not express survivin above the cut-off value. In their study, weak focal expression was also observed in the normal surrounding tissue, which is consistent with our results. However, they did not observe any expression of survivin in stromal cells. Several studies have shown significantly more frequent expression of survivin in tumor cells. For example, Tanaka et al. [29] demonstrated the expression of survivin in up to 70.7% (118/167) of breast cancers of histological stages I to III. Despite this, they did not observe any expression in the adjacent normal tissue, which is also not consistent with the results of our work. The same finding is described by Nassar et al. [30] who reported survivin expression in 84% (57/68) of mammary carcinomas while normal breast tissue was immunonegative. Shlyakhtunov and Klopova [31] determined survivin expression in 47 breast cancer samples representing 70.15% (47/67). Adamkov et al. [32] confirmed the expression in 107 cases of carcinomas out of a total of 153 (69.9%) and in another study survivin was expressed only in 55/64 (85.9%) cases of lobular carcinoma [33]. In our study, in the case of invasive breast cancer, expression was observed in 50% of cases (8/16), which is not consistent with the results of Al-Joudi et al. [34] who observed survivin expression in 68.1% (260/382) of invasive cancers. Likewise, our results in the case of CIS do not agree with other studies (only 38.5% positive samples). Okumura et al. [35] observed the cytoplasmic activity of survivin in 55.8% (29/52) of cases. Equally, Chade et al. [36] reported survivin expression in up to 75.6% (28/37) of in situ cancers. However, they observed a significant difference when comparing the grade of histopathology. In the case of low-grade CIS, only 46.2% (6/13) cases showed positive expression while up to 22 high-grade CIS out of 24 cases showed positive immunostaining for survivin. Based on their results we can assume that tumor aggressiveness is directly proportional to survivin expression. Barnes et al. [37] monitored survivin expression in patients with CIS and also with invasive breast cancer. Survivin expression was observed in 66.7% (60/92) of CIS cases and 58.6% (34/58) of IC cases. We observed that 76% (19/25) of IC and 58.3% (14/24) of CIS were positive after treatment with PTX, and 88.9% (8/9) of IC and 92% (23/25) of CIS were positive after treatment with DOX. The statistical analysis showed that IC tumors treated with paclitaxel were significantly different from non-treated tumors (*p* < 0.05) while IC tumors were not statistically significantly different. On the other hand, statistical comparison of CIS doxorubicin-treated and untreated tumors showed statistical significance (*p* < 0.05). Only a small number of studies have looked at the development of breast cancer cell resistance to paclitaxel. Kreger et al. [38], based on their study on the MDA-MB-231 cell line, hypothesized that drugs that disrupt normal microtubule dynamics cause the formation of exosomes uniquely enriched in survivin. These vesicles can then be transferred to other tumor or normal cells that form the microenvironment of the tumor and thus promote their survival. Consequently, exosomes play an important role in mediating resistance to PTX. On the other hand, a strong decrease in survivin expression in breast tumors after chemotherapy was observed by Sanchez-Rovira et al. [39]. Patients were treated with adriamycin/paclitaxel in combination with gemcitabine. The result was a reduction in proliferating cells after treatment. In our study, 67.3% positive tumors were found in the paclitaxel group (*p* < 0.05). From these results, it can be deduced that increased survivin expression is a response to PTX treatment. Our conclusion is consistent with the results of He et al. [40] who examined the sensitivity of gastric tumor cells to PTX treatment. The overall efficacy of paclitaxel was 55.56% (30/54) and lower survivin levels correlated with a better response to paclitaxel chemotherapy. Similar to PTX, acquired chemoresistance is an obstacle to the successful treatment of cancer with doxorubicin. Chang et al. [41] examined survivin expression in 33 cases of cholangiocarcinoma. A positive immunoreaction was observed in 24 of 33 cases (72.7%). No expression was observed in neighboring non-tumor cells. They also used the QBC939 cell line, where they observed higher doxorubicin-induced survivin expression. Nestal de Moraes et al. [42] observed the opposite result, noting a decrease in survivin levels in MCF-7 and MDA-MB-231 cell lines after doxorubicin exposure. At the same time, they did not observe any effect on the sensitivity of cells to DOX after inhibition of survivin. In contrast, in the osteosarcoma cell line MG63, Zhang et al. [43] demonstrated an increase in survivin expression in DOX-resistant cells. They also found that combination treatment with the survivin inhibitor YM155 and DOX significantly inhibited cell proliferation by inducing apoptosis. In this study, after an overall evaluation of the doxorubicin-treated group of animals, up to 91.20% of the samples were positive for survivin (*p* < 0.05). These results suggest that survivin may be a significant marker of established drug resistance and clinical regulation of its activity may be an effective means of reversing this resistance. Several types of survivin inhibitors are already known but in preclinical studies only. Some inhibitors have also been administered at the clinical level but the second phase of the clinical trial has not been surpassed. The possibility of targeted therapy using survivin inhibitors is a great challenge for the future as the regulation of aberrant survivin activity represents great potential for improving cancer therapy. In relation to our previous results [44] and recent data, we can conclude that the increased expression of βIII-tubulin, CA IX, and survivn is associated, positively or negatively, with PTX and DOX resistance.

## 4. Materials and Methods

### 4.1. Experimental Model

Sprague-Dawley (SD) rats are DMBA-sensitive (7,12-dimethylbenz[a]anthracene). The susceptibility peak is at 55–60 days of age. After metabolic activation in the mammary gland, the carcinogen metabolites interact with rapidly proliferating cells in the terminal end buds, forming subsequent mutations that, in turn, play a role in their transformation into malignant cells [45]. Breast cancer in female SD rats was induced by DMBA (D3254 Sigma-Aldrich, Darmstadt, Germany) administered directly to the stomach. It was prepared by dissolving 10 mg of DMBA in 1 mL of warm corn oil. It was administered in 3 doses on the 45th, 50th, and 55th postnatal days (30 mg total dose). We used 44 animals, which were kept in the standard vivarium at a temperature of 23–25 °C, relative humidity of 60–70%, and an artificial light-dark cycle of 12 h each. The rats were fed a normal diet and given tap water to drink *ad libitum*. Three to six animals were placed in one cage. During the experiment, the rats were palpated and weighed weekly. After 4–6 weeks, the first tumors appeared in all rats. The number of tumors in each animal differed from only one up to twelve tumors, averaging 3 to 6 tumors per animal. Finally, we found 125 separated tumors in 44 rats. The experiment was conducted in two stages with different animals. In the first stage, the animals were treated with paclitaxel, and doxorubicin was administered in the second stage. Rats were randomly divided into a control (physiological saline) and a paclitaxel or doxorubicin group. Rats in the paclitaxel group (n = 20) were treated with 6 doses of intraperitoneal paclitaxel (5 mg/kg). The doxorubicin group (n = 10) of female rats with tumors was treated with 6 doses of intraperitoneal doxorubicin (5 mg/kg). These rats received the first dose on the day the first tumor appeared. The next dose was given every 3–4 days. Altogether they were given 6 doses. Untreated groups (n = 07 in both cases) served as a control group that received the same amount of physiological saline instead of treatment. Four days after the last dose the animals were euthanized by an anesthetic overdose (lethal dose of Zoletil inj. −10 mg/kg). Mammary tumors were immediately excised and samples were taken for histopathological and IHC examination.

### 4.2. Immunohistochemistry (IHC)

Formalin-fixed, paraffin-embedded tissue sections from 44 female rat mammary tumors were IHC analyzed for expression of ßIII-tubulin, CA IX, and survivin proteins (ßIII-tubulin polyclonal antibody—BIOSS Antibodies, USA; CA IX polyclonal antibody—BIOSS Antibodies, USA; survivin monoclonal antibody, clone 12C4, Dako, Carpinteria, CA, USA).

Deparaffinization, rehydration, and antigen retrieval were performed on PT Link (Dako PT100) using the EnVision™ FLEX Target Retrieval Solution, at low pH 6.0 (ßIII-tubulin, survivin) and high pH 9.0 (CA IX). Following incubation in 3% H_2_O_2_ for 10 minutes, the specimens were incubated with primary antibodies. Antibody ßIII-tubulin was concentrated in a ratio of 1:200 and 1:350 for CA IX, leaving them for 30 minutes at room temperature. Sections with survivin at a 1:50 dilution were incubated for 60 minutes at room temperature.

Specimens were then incubated with the EnVision™ FLEX+ Rabbit (ßIII-tubulin, CA IX)/Mouse (survivin) LINKER for 15 min at room temperature followed by incubation with a Universal LSAB2 KIT/HRP visualization reagent for 20 min at room temperature. The enzymatic conversion of the subsequently added EnVision Flex 3,3′-diaminobenzidine tetrahydrochloride (DAB) for 5 min at room temperature resulted in the precipitation of a visible reaction product at the site of antigen. The specimens were then counterstained with hematoxylin and embedded in Pertex. All the described steps were followed by buffer washes for 5 min (EnVision^TM^ FLEX Wash Buffer 20×). Each IHC run contained a positive control and a negative antibody control (buffer, no primary antibody).

We used two methods for IHC evaluation: the semiquantitative method and the Image J Software package accompanied by the IHC profiler Plugin developed by Varghese et al. [46]. Images from random fields were captured at 40x magnification using a camera (Leica ICC50 HD) attached to a light microscope (Leica DM500).

The semiquantitative immunohistochemical method was evaluated according to the following scale:

– (minus) = 0% of positive cells (no positivity).
- 1+ = 1–10% of positive cells (weak positivity).
- 2+ = 11–90% of positive cells (intermediate positivity).
- 3+ = 91–100% of positive cells (strong positivity).

Samples marked as − (minus) and 1+ were considered as negative, samples marked as 2+ and 3+ were considered as positive.

### 4.3. Statistical Analysis

We used the “Pearson’s chi-square” (χ^2^) test to calculate statistical significance. A significance limit of 5% (*p* = 0.05) was set for the p-value and the result was considered statistically significant if the *p*-value was less than 0.05.

## Figures and Tables

**Table 1:** Expression of ßIII-tubulin in invasive carcinoma (IC) and carcinoma in situ (CIS) tumor samples from non-treated and treated groups of female rats.

ßIII-Tubulin	Paclitaxel Group						Doxorubicin Group					
Samples of carcinomas					neg.	pos.					neg.	pos.
	0	1+	2+	3+	**0/1+**	**2+/3+**	0	1+	2+	3+	**0/1+**	**2+/3+**
**42, non-treated groups**												
IC	1	3	1	0	**4**	**1**	1	4	6	0	**5**	**6**
CIS	0	2	5	1	**2**	**6**	4	6	4	4	**10**	**8**
**83, treated groups**												
IC	2	1	16	6	**3**	**22**	0	1	6	2	**1**	**8**
CIS	1	4	9	10	**5**	**19**	1	0	17	7	**1**	**24**

**Table 2:** Expression of CA IX in invasive carcinoma (IC) and carcinoma in situ (CIS) tumor samples from non-treated and treated groups of female rats.

CA IX	Paclitaxel Group						Doxorubicin Group					
Samples of carcinomas					neg.	pos.					neg.	pos.
	0	1+	2+	3+	**0/1+**	**2+/3+**	0	1+	2+	3+	**0/1+**	**2+/3+**
**42, non-treated groups**												
IC	0	3	0	2	**3**	**2**	1	6	4	0	**7**	**4**
CIS	2	2	3	1	**4**	**4**	0	7	10	1	**7**	**11**
**83, treated groups**												
IC	0	2	15	8	**2**	**23**	0	1	7	1	**1**	**8**
CIS	1	8	7	8	**9**	**15**	0	6	18	1	**6**	**19**

**Table 3:** Expression of survivin in invasive carcinoma (IC) and carcinoma in situ (CIS) tumor samples from non-treated and treated groups of female rats.

Survivin	Paclitaxel Group						Doxorubicin Group					
Samples of carcinomas					neg	pos					neg	pos
	0	1+	2+	3+	**0/1+**	**2+/3+**	0	1+	2+	3+	**0/1+**	**2+/3+**
**42, non-treated groups**												
IC	1	3	1	0	**4**	**1**	0	4	6	1	**4**	**7**
CIS	2	3	2	1	**5**	**3**	6	5	3	4	**11**	**7**
**83, treated groups**												
IC	1	5	5	14	**6**	**19**	0	1	5	3	**1**	**8**
CIS	3	7	12	2	**10**	**14**	0	2	19	4	**2**	**23**

**Table 4:** Overall expression of markers in breast cancer tumor samples from non-treated (NT) and treated (T) groups of female rats.

	Paclitaxel Group			Doxorubicin Group		
**Marker/Group**	**n**	**Posit. n (%)**	***p* Value**	**n**	**Posit. n (%)**	***p* Value**
**ßIII-tubulin**						
NT	13	7 (53.8)	*p* < 0.05	29	14 (48.3)	*p* < 0.05
T	49	41 (83.7)		34	32 (94.1)	
**CA IX**						
NT	13	6 (46.2)	*p* < 0.05	29	15 (51.7)	*p* < 0.05
T	49	38 (77.6)		34	27 (79.4)	
**Survivin**						
NT	13	4 (30.8)	*p* < 0.05	29	14 (48.3)	*p* < 0.05
T	49	33 (67.3)		34	31 (91.2)	

## Data Availability

Data available at: Department of Anatomy, Medical Faculty, Šafárik University, Šrobárova 2, 041 83 Košice, Slovakia; Alena Pastornická, alena.pastornicka@student.upjs.sk.
